# Decoration of multi-walled carbon nanotubes by polymer wrapping and its application in MWCNT/polyethylene composites

**DOI:** 10.1186/1556-276X-7-240

**Published:** 2012-05-06

**Authors:** An-En Hsiao, Shu-Ya Tsai, Mei-Wen Hsu, Shinn-Jen Chang

**Affiliations:** 1 Material and Chemical Research Laboratories, Industrial Technology Research Institute, Hsinchu, 31040, Taiwan

**Keywords:** CNTs, Polymer wrapping, Composites

## Abstract

We dispersed the non-covalent functionalization of multi-walled carbon nanotubes (CNTs) with a polymer dispersant and obtained a powder of polymer-wrapped CNTs. The UV–vis absorption spectrum was used to investigate the optimal weight ratio of the CNTs and polymer dispersant. The powder of polymer-wrapped CNTs had improved the drawbacks of CNTs of being lightweight and difficult to process, and it can re-disperse in a solvent. Then, we blended the polymer-wrapped CNTs and polyethylene (PE) by melt-mixing and produced a conductive masterbatch and CNT/PE composites. The polymer-wrapped CNTs showed lower surface resistivity in composites than the raw CNTs. The scanning electron microscopy images also showed that the polymer-wrapped CNTs can disperse well in composites than the raw CNTs.

## Background

Carbon nanotubes (CNTs), having excellent electrical and extraordinary mechanical properties, are suitable to serve as a conductive filler or a strengthening material in polymer composites; however, their chemical inertia and smooth surface result in their lack of solubility and poor compatibility with polymers. In addition, the CNTs have high aspect ratio and easily attract or tangle to each other due to the van der Waals force interaction. Furthermore, CNTs are inherently lightweight, occupy a lot of space, and are easily blown, thereby increasing the trouble in handling and the difficulty in processing and application.

To obtain good dispersion of CNTs in polymers, we can prepare the CNT masterbatch by mixing CNTs and carriers first, and then the masterbatch are diluted with thermoplastics to produce the CNT/polymer composites. There are three kinds of methods to prepare the CNT masterbatch. The first method is so-called *in situ* polymerization method [1]‐[4]: the monomers and CNTs are mixed well in a solution. Then, the monomers are polymerized, and therefore, the CNTs can disperse in the polymer polymerized from the monomers. However, this method usually needs the chemical modification of CNTs for well-dispersed CNTs in the solution, and it could damage the structure of CNTs and reduce their conductivity. The second method is the solution process [5]‐[7]: the CNTs and the polymer solution are mixed well, and the CNT/polymer composites are obtained by re-precipitation or removing the solvent from the mixture. This method can easily disperse the CNTs in the polymer; however, it is not suitable for mass production due to its high cost, toxicity of the solvent, and solubility limitations of the polymer in the solvent. The third method is the melt-mixing [5,8]: the CNTs and polymer are directly mixed at high temperature (usually above the glass transition temperature of the polymer) by mechanical mixing. The melt-mixing is also an easy way to produce the CNT masterbatch, but the CNTs are difficult to disperse well in the polymer.

Non-covalent functionalization of CNTs by polymer wrapping is a feasible process to disperse CNTs in the solvent and that could not cause dramatic changes in the electronic properties of CNTs. It has been reported that a molecular structure containing functional groups can effectively absorb on the surface of CNTs and provide the dispersion of CNTs in a solution [9]‐[16]. However, preparing the polymer-wrapped CNTs by *in situ* polymerization is not suitable to be used in CNT/polymer composites due to the residual catalysts and impurities [9]‐[11]. The surface modification of CNTs with strong acids is usually used to improve the dispersion of CNTs in polar solvents (such as H_2_O, ethanol, and DMF), but that could also disrupt the *sp*^2^ structure and conjugation of the CNTs [12]‐[14]. The CNTs functionalized with hydrophilic molecules can disperse in polar solvents, but they could not disperse well in less polar solvents (such as toluene and n-hexane) and have poor compatibility with aliphatic polymers [10]‐[16].

In this study, we reported an easy way to disperse CNTs in solvent and polymer. The surface functionalization of CNTs with a polymer dispersant, containing both aromatic group and amine group, can disperse CNTs in less polar solvents. The powder of polymer-wrapped CNTs can also re-disperse in organic solvents and be easily used in producing the CNT/polyethylene (PE) composites by melt-mixing.

## Methods

### Materials and instrumentations

Surface resistivity was measured on a surface resistivity meter, which was our homemade equipment and calibrated against indium tin oxide on PET as reference samples. The ultraviolet–visible (UV–vis) absorption spectra were recorded using a UV–vis spectrometer (SHIMADZU, Kyoto, Japan). The CNT/PE composites were prepared using a twin-screw extruder (MP-2015, APV, Houston, TX, USA).

All other chemicals which were not specially mentioned were commercially available and used as supplied. We used the multi-walled carbon nanotubes purchased from Nanomaterial Store (OD, 10 to 30 nm; length, 10 to 30 μm; purity, 85 %; Fremont, CA, USA). The polymer dispersant, which was synthesized form the monomers of phenyl methacrylate and glycidyl methacrylate and followed by grafting with amine, had a weight-average molecular weight of about 10,000 and an amine value (mg KOH) of 14.

### Preparation of the polymer-wrapped CNTs

The polymer dispersant, CNTs, and solvent were added to a reactor, and they were mixed by ultrasonic oscillation and mechanical stirring for 1 h to form the CNT suspension. After filtering the CNT suspension and collecting the sample, the sample was baked using an oven to obtain the powder of polymer-wrapped CNTs.

### Preparation of the CNT masterbatch and CNT/PE composites

The powder of polymer-wrapped CNTs and PE were blended using a kneader first, and then blended using a twin-screw extruder to form the 10 wt.% CNT masterbatch. The CNT masterbatch was further diluted with PE using the kneader and twin-screw extruder to form the CNT/PE composites with various concentrations of CNTs.

## Results and discussions

### Dispersion of the powder of polymer-wrapped CNTs

**Figure 1:**
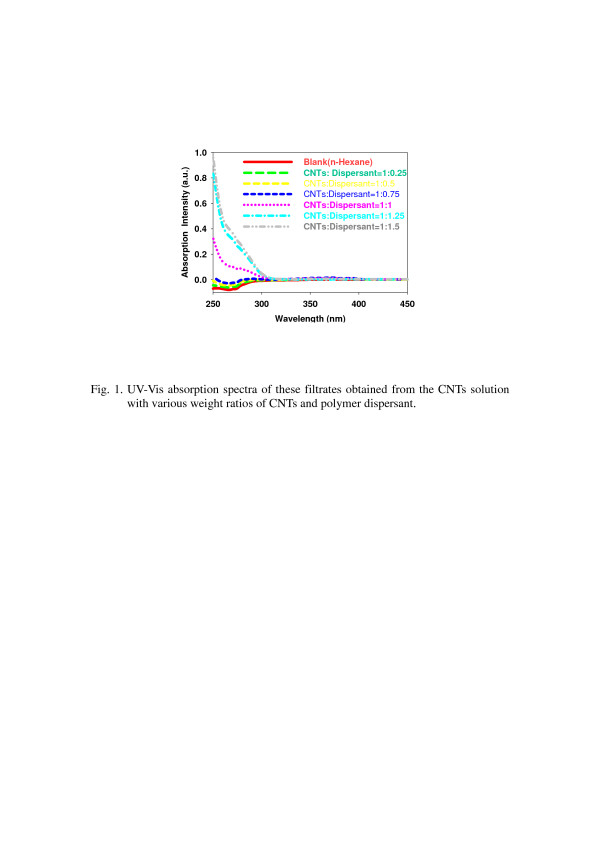
**UV–vis absorption spectra of filtrates obtained from the CNT solutions.** The solutions have various weight ratios of CNTs and polymer dispersant.

**Figure 2:**
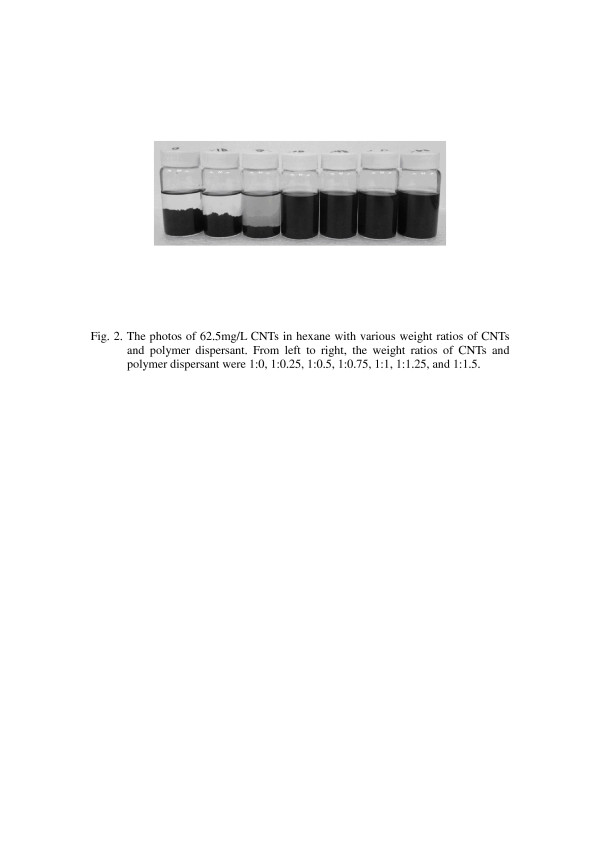
**Photo of 62.5-mg/L CNTs in hexane with various weight ratios of CNTs and polymer dispersant.** From left to right, the weight ratios of CNTs and polymer dispersant were 1:0, 1:0.25, 1:0.5, 1:0.75, 1:1, 1:1.25, and 1:1.5.

Adding too much polymer dispersant will keep excessive polymer dispersant in composites, and it could result in composites with a poor mechanical property. To determine the optimal additive amount of the polymer dispersant for an effective polymer wrapping, we prepared the dispersion of CNTs in n-hexane with various additive amounts of polymer dispersant, filtered the dispersion with a 0.25-μm filter to remove the CNTs, and measured the UV absorption bands of these filtrates. The UV–vis absorption spectra were shown in Figure 1. The absorbing bands at wavelengths of 250 to 300 nm increased with increasing additive amounts of the polymer dispersant because of the increase of polymer dispersants containing phenyl groups in the filtrate, and it meant that the polymer dispersant could be in the filtrate when the weight ratios of CNTs and polymer dispersant were above 1:0.75. We suggested that the optimal weight ratio of CNTs and polymer dispersant, which can sufficiently wrap around CNTs, was 1:0.75, and it can also be proved by preparing the stable CNT dispersion when the weight ratios of CNTs and polymer dispersant were above 1:0.75 as shown in Figure 2. The highest concentration of CNTs in the solution in which we had tried to prepare the polymer-wrapped CNTs was up to 2.5 wt.%, which can also disperse well in the solution. Nevertheless, it is noteworthy that the more CNTs added, the higher the viscosity of the solution was. We needed to increase the dispersion time and adjust the stirring speed for the good dispersion of CNTs.

**Figure 3:**
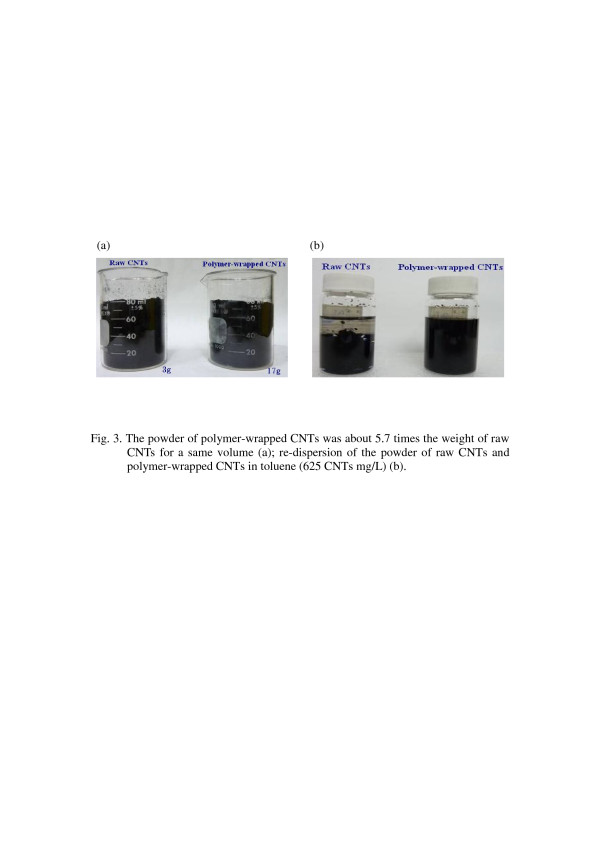
**The powder of raw and polymer-wrapped CNTs and their re-dispersion in toluene.** (**a**) The powder of polymer-wrapped CNTs was about 5.7 times the weight of raw CNTs for the same volume. (**b**) Re-dispersion of the powder of raw and polymer-wrapped CNTs in toluene (625-mg/L CNTs).

**Figure 4:**
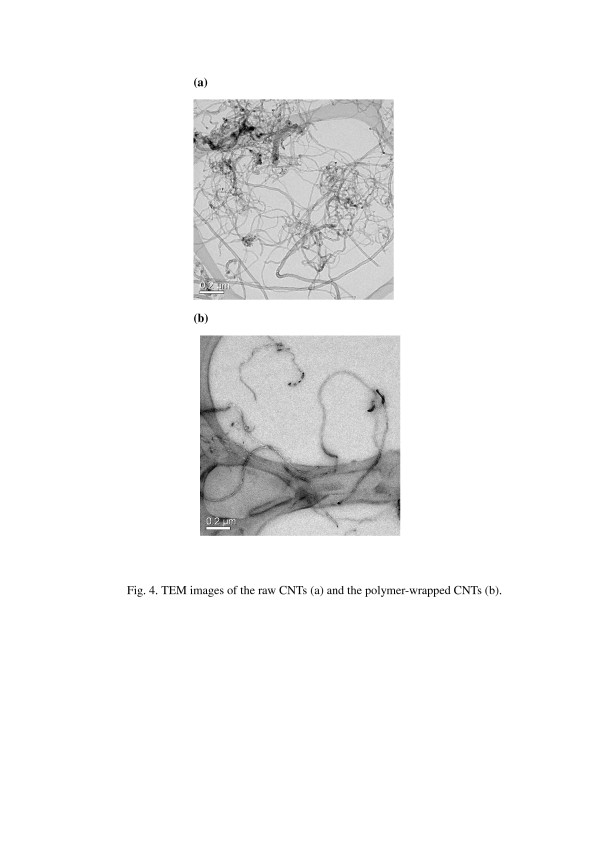
TEM images of (a) the raw CNTs and (b) the polymer-wrapped CNTs.

In the following experiments, the weight ratio of the CNTs and polymer dispersant we have chosen was 1:0.75 to prepare the polymer-wrapped CNTs. The raw CNTs had these properties of being lightweight and difficult to disperse in a solution; however, the polymer-wrapped CNTs was about 5.7 times (about 4.4 times while taking off the weight of the polymer dispersant) the weight of raw CNTs for the same volume as shown in Figure 3a, and the polymer-wrapped CNTs can also re-disperse in toluene as shown in Figure 3b. Transmission electron microscopy (TEM) was performed to reveal the structures of the raw CNTs and polymer-wrapped CNTs dispersed in toluene. In Figure 4a, the raw CNTs had the tendency to aggregate severely due to strong van der Waals attractions among CNTs, and the diameters of these bundles typically ranged from tens to hundreds of nanometers. On the contrary, the polymer dispersant which functionalized the surface of CNTs can prevent them from aggregation, and individual CNTs can be observed as shown in Figure 4b.

### MWCNT/PE composites

**Figure 5:**
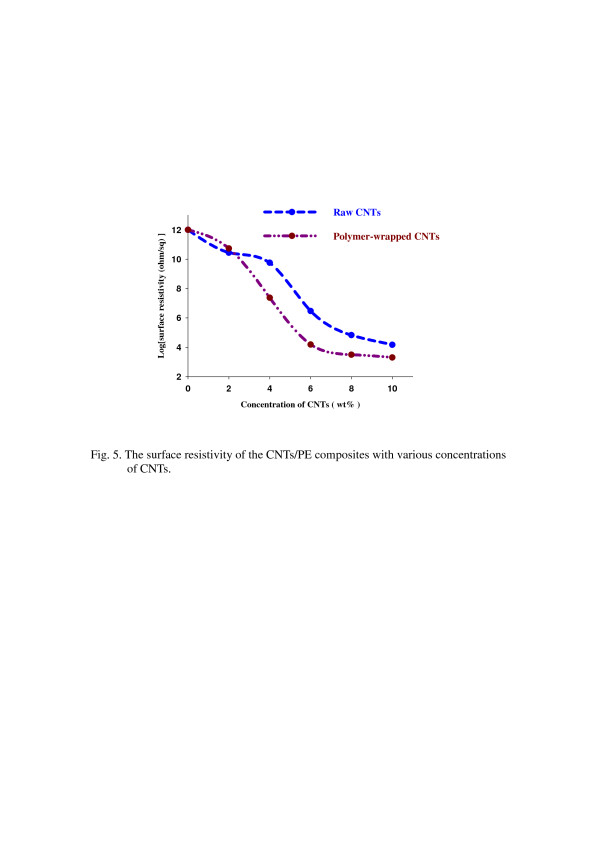
The surface resistivity of the CNT/PE composites with various concentrations of CNTs.

The surface resistivity of CNT/PE composites with various concentrations of CNTs was shown in Figure 5. It was apparent that the polymer-wrapped CNTs can offer a lower surface resistivity than the raw CNTs. It indicated that the polymer-wrapped CNTs could disperse well in PE and easily form more conductive networks in composites. It has been reported that the more conductive networks in composites, the better the conductivity [17,18]. The surface resistivity dropped sharply when the concentrations of CNTs were above 2 wt.%. It was believed that more conductive networks were formed simultaneously at the critical concentration of CNTs [17,18]. The surface resistivity seemed to drop gradually as the concentrations of CNTs were above 7 wt.% and also indicated that the conductive networks were about to be saturated. Because the raw CNTs had poor dispersion and tended to aggregate in the polymer, they could more easily form major conducting channels than the polymer-wrapped CNTs, and therefore, we observed that the raw CNTs showed lower surface resistivity than the polymer-wrapped CNTs before the critical concentration. However, after the critical concentration, the polymer-wrapped CNTs showed a lower surface resistivity than the raw CNTs because the polymer-wrapped CNTs could disperse well in the polymer and form more effective conductive networks than the raw CNTs. Finally, we observed that the polymer-wrapped CNTs revealed a lower surface resistivity.

**Figure 6:**
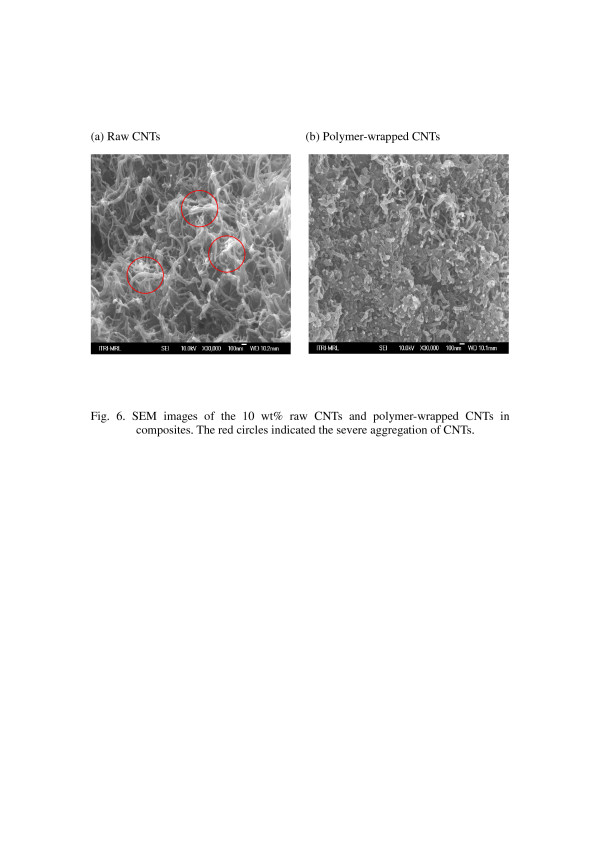
**SEM images of the (a) 10 wt.% raw CNTs and (b) polymer-wrapped CNTs in composites.** The red circles indicate the severe aggregation of CNTs.

To understand the difference in surface resistivity between the raw CNTs and the polymer-wrapped CNTs in composites, we observed the dispersion of CNTs in composites by scanning electron microscopy (SEM). A smaller diameter of the polymer-wrapped CNTs with respect to the raw CNTs was observed in Figure 6. It was obvious that the raw CNTs presented a larger tendency to form small bundles, and the diameters of these bundles ranged from tens to hundreds of nanometers. On the contrary, the polymer-wrapped CNTs had a smaller tendency to form small bundles and exhibited good compatibility with PE. The result is that we can obtain a lower surface resistivity for the good dispersion of CNTs in composites.

## Conclusion

Decoration of CNTs by polymer wrapping is an easy way to disperse CNTs in solvent and polymer, and the powder of polymer-wrapped CNTs can re-disperse in a solvent. This technology also improves the drawbacks of CNTs of being lightweight and difficult to process. Depending on the concentration of CNTs in composites, they can meet electrical requirements such as anti-electrostatic property $\left(10^{9}–10^{12}Ω/\mathrm{sq}\right)\text{,}$ electrostatic discharge $(10^{6}–10^{9}Ω/\mathrm{sq})\text{,}$ conductivity $(≦10^{6}Ω/\mathrm{sq})\text{,}$ and electromagnetic interference $(≦10^{4}Ω/\mathrm{sq})\text{.}$ The composites can be applied in an anti-electrostatic product, an electrostatic discharge product, an electromagnetic and radiation shield, and 3 C electronic equipment.

## Competing interests

The authors declare that they have no competing interests.

## Authors' contributions

AEH conceived the study, coordinated the research, and drafted the manuscript. SJC and SYT participated in the study of molecular structures and the data analysis. MWH carried out the sample preparation and the experimental measure. All authors did the analysis and interpretation of experimental data. All authors read and approved the final manuscript.
